# Danish and British dementia *ITM2b/BRI2* mutations reduce BRI2 protein stability and impair glutamatergic synaptic transmission

**DOI:** 10.1074/jbc.RA120.015679

**Published:** 2020-11-22

**Authors:** Tao Yin, Wen Yao, Alexander D. Lemenze, Luciano D’Adamio

**Affiliations:** 1 Department of Pharmacology, Physiology & Neuroscience, New Jersey Medical School, Brain Health Institute, Jacqueline Krieger Klein Center in Alzheimer's Disease and Neurodegeneration Research, Rutgers, The State University of New Jersey, Newark, New Jersey, USA; 2 Department of Pathology, Immunology, and Laboratory Medicine, New Jersey Medical School, The State University of New Jersey, Newark, New Jersey, USA

**Keywords:** neurodegenerative disease, synaptic plasticity, mouse, animal model, glutamate, protein turnover, EMEM, Eagle's Minimum Essential Medium, ERLAD, ER-to-lysosomes-associated degradation, FBD, familial British dementia, FDD, familial Danish dementia, ISI, interstimulus interval, KI, knock-in, LTP, long-term potentiation, mEPSC, miniature excitatory postsynaptic currents, PPF, paired-pulse facilitation, PPR, paired-pulse ratio

## Abstract

Mutations in *integral membrane protein 2B* (*ITM2b/BRI2*) gene cause familial British and Danish dementia (FBD and FDD), autosomal dominant disorders characterized by progressive cognitive deterioration. Two pathogenic mechanisms, which may not be mutually exclusive, have been proposed for FDD and FBD: 1) loss of BRI2 function; 2) accumulation of amyloidogenic mutant BRI2-derived peptides, but the mechanistic details remain unclear. We have previously reported a physiological role of BRI2 in excitatory synaptic transmission at both presynaptic termini and postsynaptic termini. To test whether pathogenic *ITM2b* mutations affect these physiological BRI2 functions, we analyzed glutamatergic transmission in FDD and FBD knock-in mice, which carry pathogenic FDD and FBD mutations into the mouse endogenous *Itm2b* gene. We show that in both mutant lines, spontaneous glutamate release and AMPAR-mediated responses are decreased, while short-term synaptic facilitation is increased, effects similar to those observed in *Itm2b*^*KO*^ mice. *In vivo* and *in vitro* studies show that both pathogenic mutations alter maturation of BRI2 resulting in reduced levels of functional mature BRI2 protein at synapses. Collectively, the data show that FDD and FBD mutations cause a reduction of BRI2 levels and function at synapses, which results in reduced glutamatergic transmission. Notably, other genes mutated in Familial dementia, such as *APP*, *PSEN1*/*PSEN2*, are implicated in glutamatergic synaptic transmission, a function that is altered by pathogenic mutations. Thus, defects in excitatory neurotransmitter release may represent a general and convergent mechanism leading to neurodegeneration. Targeting these dysfunction may offer a unique disease modifying method of therapeutic intervention in neurodegenerative disorders.

*ITM2b/BRI2* gene is a gene that codes a type II membrane protein called BRI2. The immature BRI2 precursor (imBRI2) is cleaved at the C-terminus by proprotein convertase to produce the mature NH_2_-terminal BRI2 (mBRI2) and a COOH-terminal peptide of 23 amino acid called Bri23 (1). Two autosomal dominant mutations in *ITM2b/BRI2* cause FDD and FBD. The FDD mutation consists of a 10-nucleotide duplication one codon before the normal stop codon (2). This produces a frameshift in the BRI2 sequence generating a precursor protein 11 amino acids larger than normal. In the British kindred, a point mutation at the stop codon of *BRI2* results in a read-through of the 3’-untranslated region and the synthesis of a BRI2 molecule containing 11 extra amino acids at the COOH-terminus (3). Convertase-mediated cleavage of immature mutant Danish and British BRI2 generates two distinct 34 amino acid long peptides, called ADan and ABri, respectively. Both peptides are deposited as amyloid fibrils. For clarity, we will refer to the wild-type imBRI2 as BRI2-Bri23, to the Danish mutant imBRI2 as BRI2-Adan, and to the British mutant imBRI2 as BRI2-ABri. Since all three imBRI2 proteins form the same mBRI2 polypeptide, all mBRI2 proteins will be referred to as mBRI2.

Using a genetic approach to inactivate *Itm2b* in either presynaptic (CA3), or postsynaptic (CA1), or both (CA3+CA1) neurons of the hippocampal Schaeffer-collateral pathway (SC–CA3>CA1 synapses) in mice, we found a physiological role of Bri2 in the excitatory synaptic transmission (4). Loss of Bri2 impairs glutamatergic neurotransmitter release and AMPAR-mediated responses in SC–CA3>CA1 synapses by a presynaptic and postsynaptic mechanism, respectively. This evidence suggests that Bri2 facilitates glutamate transmission *via* both presynaptic and postsynaptic mechanism. Given the relevance of excitatory synaptic transmission to cognition and that BRI2 mutations cause neurodegeneration and dementia in humans, we tested whether the pathogenic Danish and British dementia mutations will alter, one way or another, this physiological function of BRI2. To test this hypothesis, we studied glutamatergic neurotransmission in SC–CA3>CA1 synapses of mice carrying the pathogenic Danish and British dementia mutations (*Itm2b*^*D/D*^ and *Itm2b*^*B/B*^ mice) into the *Itm2b* mouse gene (5, 6, 7). We choose a knock-in (KI) approach because: a) KIs mimic familial dementia genetics and make no assumption about pathogenic mechanisms; b) expression of mutant genes is controlled by endogenous regulatory elements, thereby allowing us to test the hypothesis in a biologically relevant model organism. This strategy allows examination of the effects of pathogenic *Itm2b* mutations on the function of Bri2 in excitatory synaptic transmission on both presynaptic and postsynaptic neurons of the Schaeffer-collateral pathway.

## Results

### Pathogenic Bri2 mutations impair glutamatergic transmission at excitatory hippocampal SC–CA3>CA1 synapses

**Figure 1:**
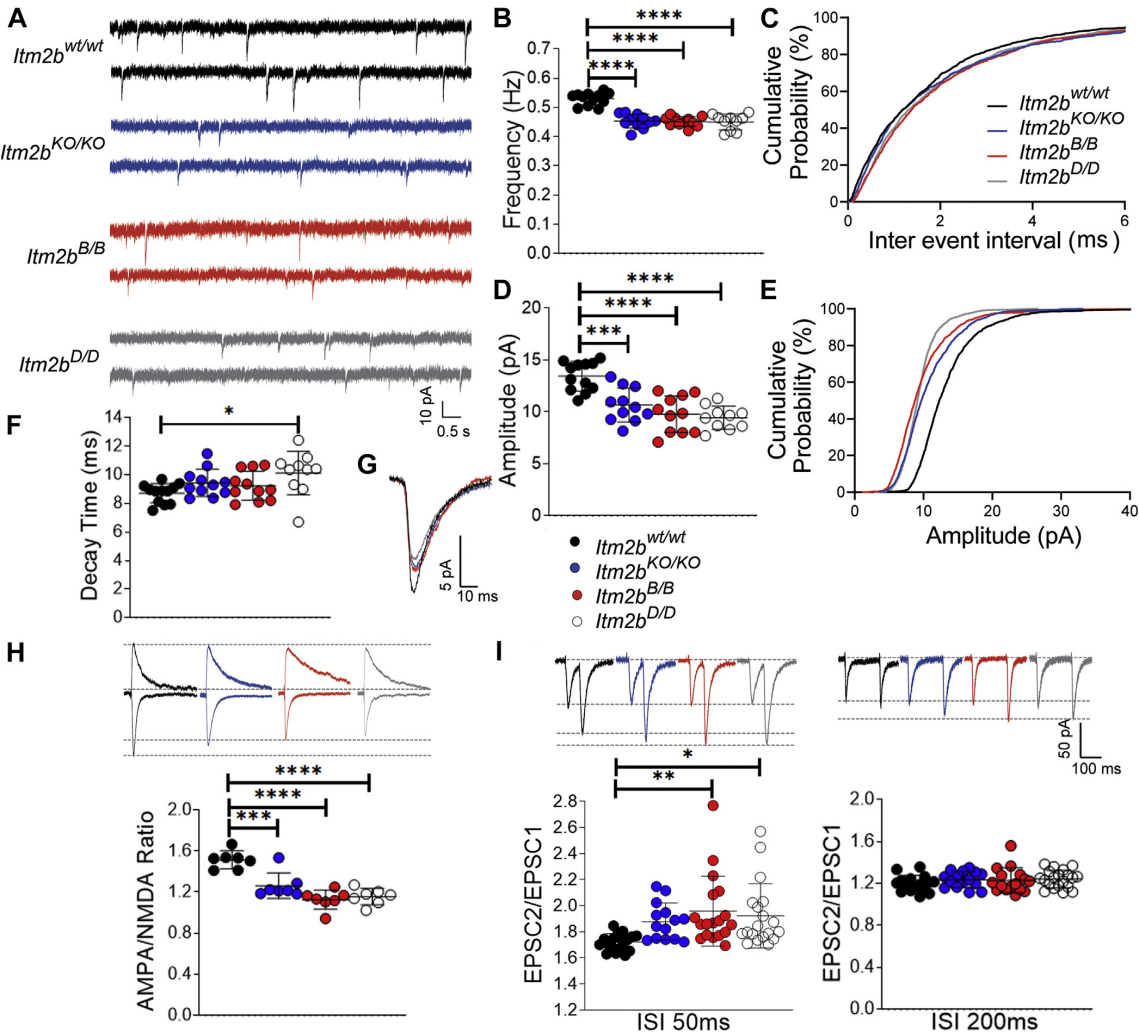
**The Danish and British mutations reduce glutamatergic synaptic transmission at hippocampal SC–CA3>CA1 synapses.***A*, representative recording traces of mEPSC at SC–CA3>CA1 pyramidal cell synapses. *B*, the deletion of *Itm2b* (*Itm2b*^*KO/KO*^), the British and Danish mutations (*Itm2b*^*B/B*^ and *Itm2b*^*D/D*^) cause a significant decrease in mEPSC frequency [ANOVA summary, F_(3, 40)_ = 35.44, *p* < 0.0001∗∗∗∗; post-hoc Tukey's multiple comparisons test: *Itm2b*^*WT/WT*^*versus Itm2b*^*KO/KO*^, *p* < 0.0001∗∗∗∗; *Itm2b*^*WT/WT*^*versus Itm2b*^*B/B*^, *p* < 0.0001∗∗∗∗; *Itm2b*^*WT/WT*^*versus Itm2b*^*D/D*^, *p* < 0.0001∗∗∗∗; *Itm2b*^*−/−*^*versus Itm2b*^*B/B*^, *p* = 0.9982 (ns); *Itm2*^*KO/KO*^*versus Itm2b*^*D/D*^, *p* = 0.9825 (ns); *Itm2b*^*B/B*^*versus Itm2b*^*D/D*^, *p* = 0.9969 (ns)]. *C*, cumulative probability of AMPAR-mediated mEPSC frequency inter event intervals. *D*, *Itm2b*^*KO/KO*^, *Itm2b*^*B/B*^ and *Itm2b*^*D/D*^ mice show a significant decrease in mEPSC amplitude [ANOVA summary, F_(3, 40)_ = 16.59, *p* < 0.0001∗∗∗∗; post-hoc Tukey's multiple comparisons test: *Itm2b*^*WT/WT*^*versus Itm2b*^*KO/KO*^, *p* = 0.0004∗∗∗; *Itm2b*^*WT/WT*^*versus Itm2b*^*B/B*^, *p* < 0.0001∗∗∗∗; *Itm2b*^*WT/WT*^*versus Itm2b*^*D/D*^, *p* < 0.0001∗∗∗∗; *Itm2*^*KO/KO-*^*versus Itm2b*^*B/B*^, *p* = 0.5223 (ns); *Itm2*^*KO/KO*^*versus Itm2b*^*D/D*^, *p* = 0.2569 (ns); *Itm2b*^*B/B*^*versus Itm2b*^*D/D*^, *p* = 0.9516 (ns). *E*, cumulative probability of AMPAR-mediated mEPSC amplitude. *F*, in contrast, decay time of mEPSC was modestly increased, but only in the *Itm2b*^*D/D*^ mice [F (_2, 30_) = 3.284, *p* = 0.0304∗; post-hoc Tukey's multiple comparisons test: *Itm2b*^*WT/WT*^*versus Itm2b*^*KO/KO*^, *p* = 3590 (ns); *Itm2b*^*WT/WT*^*versus Itm2b*^*B/B*^, *p* = 0.6339 (ns); *Itm2b*^*WT/WT*^*versus Itm2b*^*D/D*^, *p* = 0.0176∗; *Itm2*^*KO/KO-*^*versus Itm2b*^*B/B*^, *p* = 0.9585 (ns); *Itm2b*^*KO/KO*^*versus Itm2b*^*D/D*^, *p* = 0.4707 (ns); *Itm2b*^*B/B*^*versus Itm2b*^*D/D*^, *p* = 0.2433 (ns)]. *G*, average mEPSC of the three groups depicts differences in amplitude. *H*, AMPA/NMDA ratio is significantly decreased in all three mutant mice [ANOVA summary, F (_3, 24_) = 22.73, *p* < 0.0001∗∗∗∗; post-hoc Tukey's multiple comparisons test: *Itm2b*^*WT/WT*^*versus Itm2*^*KO/KO*^, *p* = 0.0004∗∗∗; *Itm2b*^*WT/WT*^*versus Itm2b*^*B/B*^, *p* < 0.0001∗∗∗∗; *Itm2b*^*WT/WT*^*versus Itm2b*^*D/D*^, *p* < 0.0001∗∗∗∗; *Itm2b*^*KO/KO*^*versus Itm2b*^*B/B*^, *p* = 0.0679 (ns); *Itm2b*^*KO/KO*^*versus Itm2b*^*D/D*^, *p* = 0.1988 (ns); *Itm2b*^*B/B*^*versus Itm2b*^*D/D*^, *p* = 0.9416 (ns)]. Representative traces are shown on top of the graph (traces are averaged from 20 sweeps). *I*, average PPF at 50 ms (*left panel*) and 200 ms (*right panel*) Inter stimulus Interval (ISI) shows that PPF is increased in *Itm2b*^*B/B*^ and *Itm2b*^*D/D*^ mice, but only at 50 ms ISI [PPF at 50 ms ISI: ANOVA summary, F_(3, 65)_ = 4.855, *p* = 0.0041∗∗; post-hoc Tukey's multiple comparisons test: *Itm2b*^*WT/WT*^*versus Itm2b*^*KO/KO*^, *p* = 0.1358 (ns); *Itm2b*^*WT/WT*^*versus Itm2b*^*B/B*^, *p* < 0.0043∗∗; *Itm2b*^*WT/WT*^*versus Itm2b*^*D/D*^, *p* = 0.0176∗; *Itm2b*^*KO/KO*^*versus Itm2b*^*B/B*^, *p* = 0.06888 (ns); *Itm2b*^*KO/KO*^*versus Itm2b*^*D/D*^, *p* = 0.9256 (ns); *Itm2b*^*B/B*^*versus Itm2b*^*D/D*^, *p* = 0.9506 (ns); PPF at 200 ms ISI: ANOVA summary F_(3, 63)_ = 0.7463, *p* = 0.5285 (ns)]. Representative traces are shown on top of the panels. Data are represented as mean ± SD and were analyzed by ordinary one-way ANOVA followed by post-hoc Tukey's multiple comparisons test when ANOVA showed significant differences. We used n = 6 animals for each genotype, three males and three females.

*Itm2b*^*D/D*^ and *Itm2b*^*B/B*^ mice were derived as described previously (5, 6, 7). To test whether these pathogenic mutations alter the synaptic function of Bri2 in the excitatory synaptic transmission at SC–CA3>CA1 synapses (4), we compared the following genotypes: *Itm2b*^*WT/WT*^, *Itm2b*^*KO/KO*^ (mice carrying two knockout *Itm2b* alleles), *Itm2b*^*D/D*^ (mice carrying two *Itm2b* alleles with the Danish mutation), and *Itm2b*^*B/B*^ (mice carrying two *Itm2b* alleles with the British mutation). Synaptic transmission was studied in 4 to 6-month-old *Itm2b*^*WT/WT*^ (three males and three females), *Itm2b*^*KO/KO*^ (three males and three females), *Itm2b*^*D/D*^ (three males and three females), and *Itm2b*^*B/B*^ (four males and three females) mice. First, we analyzed miniature excitatory postsynaptic currents (mEPSC), the frequency of which is also determined, in part, by the probability of synaptic vesicles release (P*r*) (8): a decrease in P*r* leads to a decrease in frequency and vice versa. As previously shown (4) and in accord with the hypothesis that endogenous Bri2 facilitates glutamate release, the frequency of mEPSC was reduced in *Itm2b*^*KO/KO*^ mice (Fig. 1, *A*–*C*). The British and Danish *Itm2b* mutations acted analogously, both causing a reduction in the frequency of mEPSC (Fig. 1, *A*–*C*).

The amplitude of mEPSC is dependent on postsynaptic AMPA (α-amino-3-hydroxy-5-methyl-4-isoxazole propionic acid) receptor (AMPAR-mediated responses). Consistently with our previous report, the amplitude of mEPSC was significantly decreased in *Itm2b*^*KO/KO*^ mice (Fig. 1, *D*, *E* and *G*). Like for the mEPSC frequency, *Itm2b*^*D/D*^ and *Itm2b*^*B/B*^ mice showed a phenotype similar to *Itm2b*^*KO/KO*^ animals (Fig. 1, *D*, *E* and *G*). The decay time of mEPSC was slightly higher in all mutant lines: however, this slight increase reached statistical significance only for the *Itm2b*^*D/D*^ mice (Fig. 1*F*).

Next, we measured AMPAR and NMDAR-dependent synaptic responses. Consistent with the hypothesis that Bri2 boosts AMPAR-mediated responses, the AMPA/NMDA ratio was reduced in *Itm2b*^*KO/KO*^ mice (Fig. 1*H*). Once again, a similar decrease was observed in *Itm2b*^*D/D*^ and *Itm2b*^*B/B*^ mice (Fig. 1*H*).

Finally, we examined the effect of *Itm2b* inactivation and pathogenic mutations on paired-pulse facilitation (PPF). PPF is a form of short-term synaptic plasticity that is determined, at least in part, by changes in P*r* of glutamatergic synaptic vesicles (SV): inversely to mEPSC frequency, a decrease in P*r* leads to an increase in facilitation and vice versa (8). Previous studies on older mice showed that PPF at 50 ms interstimulus interval (ISI), but not at 200 ms ISI, was significantly increased in *Itm2b*^*KO/KO*^ mice (4). In this set of studies, facilitation at 50 ms ISI was increased in *Itm2b*^*KO/KO*^: however, this increase did not reach statistical significance (Fig. 1*I*). This discrepancy with our previous report is probably due to the lower number of recordings analyzed here. Yet, both *Itm2b*^*D/D*^ and *Itm2b*^*B/B*^ mice showed increased PPF as compared with control *Itm2b*^*WT/WT*^ animals at 50 ms ISI (Fig. 1). Overall, our data indicate that *Itm2b* inactivation and both Danish and British pathogenic *Itm2b* mutations alter glutamatergic synaptic transmission at excitatory hippocampal SC–CA3>CA1 synapses analogously.

### Reduced synaptic levels of mBri2 in *Itm2b*^*B/B*^ and *Itm2b*^*D/D*^ mice

**Figure 2:**
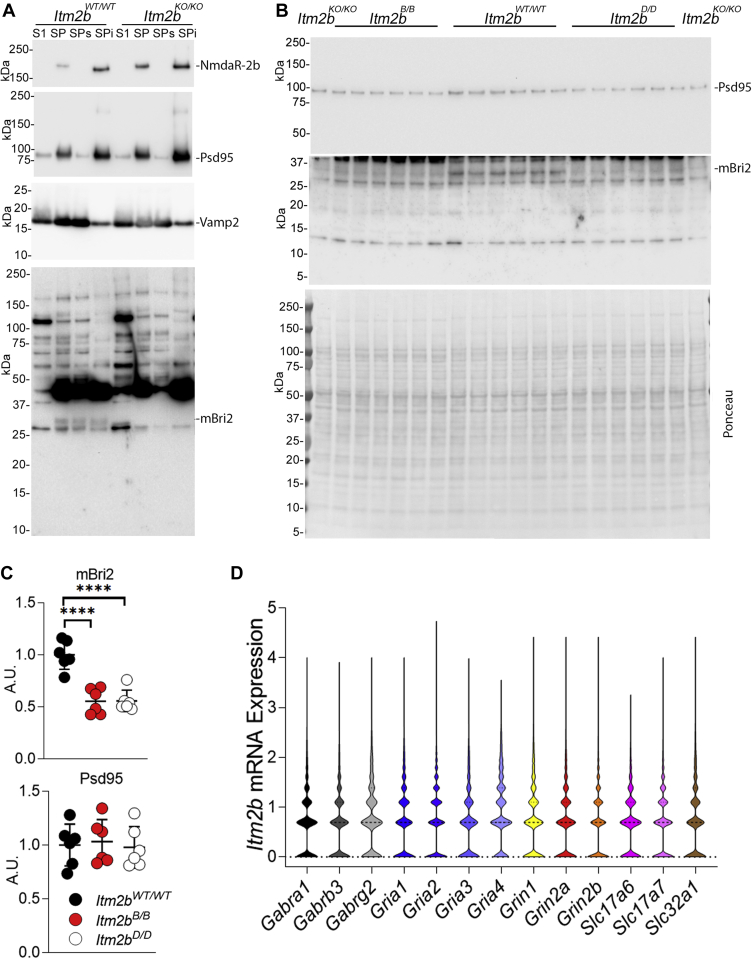
**Expression of mBri2 is reduced in synaptoneurosome fractions derived from *Itm2b***^***B/B***^**and *Itm2b***^***D/D***^**mice.***A*, synaptoneurosome fractions (SP) were separated from total brain homogenates (S1) of *Itm2b*^*WT/WT*^ and *Itm2b*^*KO/KO*^ and were further separated into triton-X soluble (SPs) and insoluble fraction (SPi). Presynaptic proteins (such as Vamp2) are enriched in SP as compared with S1 and are mainly distributed in the SPs fraction. Postsynaptic proteins (such as NmdaR-2b and Psd95) are enriched in SP as compared with S1 and are mainly distributed in the SPi fraction. These data attest the good quality of the SP preparation. Consistent with pre- and postsynaptic activity of Bri2, mBri2 is found in both pre- and postsynaptic enriched fractions. Since the anti-Bri2 antibody used in WB has many background bands, we always use *Itm2b*^*KO/KO*^ samples as a negative control to determine with certainty which band corresponds to Bri2. *B*, analysis of mBri2 and PSD95 expression in synaptoneurosomes of 4 to 6-month-old mice (we used three male and three female mice for each genotype, *Itm2b*^*WT/WT*^, *Itm2b*^*D/D*^, and *Itm2b*^*B/B*^). The red ponceau staining, which indicates the total protein loaded on the gel, of the membrane used for Western immunoblot analysis is also shown. *C*, the density of mBri2 and Psd95 was measured and normalized to red ponceau intensity. *D*, violin plot illustrating the normalized expression values of *Itm2b* across selected gene positive cells. Cells were respective gene positive with a normalized expression value greater than 1. Data are represented as mean ± SD and analyzed by ordinary one-way ANOVA followed by post-hoc Tukey's multiple comparisons test when ANOVA showed significant differences. No differences were seen in Psd95 [ANOVA summary, F (_2, 15_) = 0.1105, *p* = 0.8961 (ns)]. Significant difference was observed in mBri2 expression [ANOVA summary, F (_2, 15_) = 26.33, *p* < 0.0001∗∗∗∗, post-hoc Tukey's multiple comparisons test: *Itm2b*^*WT/WT*^*versus Itm2b*^*B/B*^, *p* < 0.0001∗∗∗∗, *Itm2b*^*WT/WT*^*versus Itm2b*^*D/D*^, *p* < 0.0001∗∗∗∗, *Itm2b*^*B/B*^*versus Itm2b*^*D/D*^, *p* > 0.05 ns].

The evidence that genetic inactivation of Bri2 expression in pre- and postsynaptic neurons reduced excitatory synaptic transmission at hippocampal SC-CA3>CA1 synapse indicates that neuronal, and perhaps synaptic, mBri2 expression is important for physiological glutamate transmission. To determine whether the synaptic impairment in *Itm2b*^*B/B*^ and *Itm2b*^*D/D*^ mice is associated with loss of mBri2 at synapses, we examined Bri2 expression in synaptoneurosome fraction (Fig. 2*A* shows the quality of synaptosomal fractions) prepared from whole brain lysates of 4 to 6-month-old mice (n = 6 for each genotype). We observed the following: 1) Bri2 is present in synaptoneurosomes (Fig. 2, *A*–*B*); 2) only mBri2 is found in synaptoneurosomes (Fig. 2, *A*–*B*), which is consistent with the evidence that only mature Bri2 transported to surface membrane (9); 3) mBri2 expression is significantly and similarly reduced in *Itm2b*^*B/B*^ and *Itm2b*^*D/D*^ mice compared with *Itm2b*^*WT/WT*^ mice (Fig. 2, *B*–*C*). These results may explain why glutamatergic synaptic transmission is similarly affected in *Itm2b*^*KO/KO*^, *Itm2b*^*B/B*^, and *Itm2b*^*D/D*^ mice.

The synaptoneurosome preparation includes synaptic elements from both excitatory and inhibitory neurons. To ascertain *Itm2b* mRNA expression in various neuronal populations, we analyzed publicly available scRNA-seq to test *Itm2b* mRNA coexpression with GABA_A_-receptor (*Gabra1*, *Gabrb3*, *Gabrg2*), AMPA-receptor (Gria1, Gria2, Gria3, Gria4), and NMDA-receptor (*Grin1*, *Grin2a*, *Grin2b*) genes. This analysis shows similar levels of coexpression of *Itm2b* with these neuronal markers (Fig. 2*D*). In addition, we determine coexpression of *Itm2b* with *Slc32a1*, which encodes for the vesicular inhibitory amino acid transporter Viaat and is a marker of inhibitory neurons, *Slc17a7* and *Slc17a6*, which encode for vesicular glutamate transporter 1 Vglut1 and vesicular glutamate transporter 2 Vglut2, respectively, and are markers of excitatory neurons. The results suggest that *Itm2b* mRNA is equally expressed in excitatory and inhibitory neurons (Fig. 2*D*). This data suggest that it will be worth, in future experiments, testing the role of Bri2 and the effects of the Danish and British mutations on GABAergic transmission as well.

### Trafficking and maturation of mutant human BRI2 proteins are altered in transfected cell line

**Figure 3:**
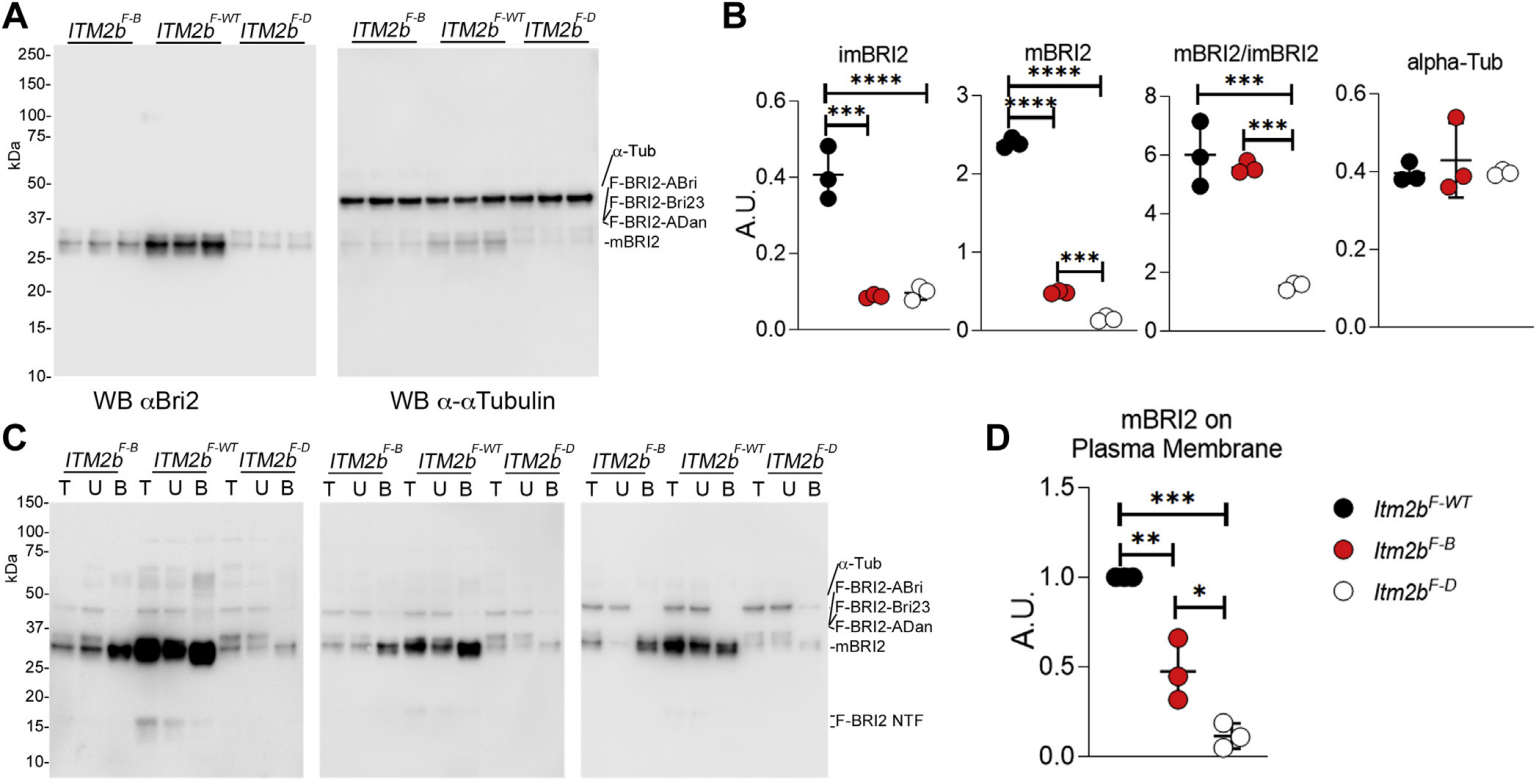
**The British and Danish mutations alter maturation and trafficking of human in transfected HeLa cells.***A*, biological replicates of HeLa cells were transfected with *ITM2b*^*F-WT*^, *ITM2b*^*F-B*^, and *ITM2b*^*F-D*^ constructs and lysed after 24 h. The level of Bri2 expression was determined by Western immunoblot analysis. We used n = 3 independent replicates for each transfection. *B*, the density of imBri2 (Bri2-ABri or Bri2-Bri23 or Bri2-ADan), mBri2, mBri2/imBri2 ratio, and α-Tubulin were measured and normalized to red ponceau intensity. Data are represented as mean ± SD and analyzed by ordinary one-way ANOVA followed by post-hoc Tukey's multiple comparisons test when ANOVA showed significant differences. No differences were seen in a-Tubulin [ANOVA summary, F _(2, 6)_ = 0.3225, *p* = 0.7361]. Significant differences were observed in both imBri2 and mBri2 expression [ANOVA summary, imBri2: F _(2, 6)_ = 57.24, *p* = 0.0001∗∗∗, post-hoc Tukey's multiple comparisons test: *ITM2b*^*F-WT*^*versus ITM2b*^*F-B*^, *p* < 0.001∗∗∗, *ITM2b*^*F-WT*^*versus ITM2b*^*F-D*^, *p* < 0.001∗∗∗, *ITM2b*^*F-B*^*versus ITM2b*^*F-D*^, *p* > 0.05 ns. mBri2: F _(2, 6)_ = 2557, *p* < 0.0001∗∗∗∗, post-hoc Tukey's multiple comparisons test: *ITM2b*^*F-WT*^*versus ITM2b*^*F-B*^, *p* < 0.0001∗∗∗∗, *ITM2b*^*F-WT*^*versus ITM2b*^*F-D*^, *p* < 0.0001∗∗∗∗, *ITM2b*^*F-B*^*versus ITM2b*^*F-D*^, *p* < 0.001∗∗∗, mBri2/imBri2: F _(2, 6)_ = 42.91, *p* = 0.0003∗∗∗, post-hoc Tukey's multiple comparisons test: *ITM2b*^*F-WT*^*versus ITM2b*^*F-B*^, *p* > 0.05 ns, *ITM2b*^*F-WT*^*versus ITM2b*^*F-D*^, *p* < 0.001∗∗∗, *ITM2b*^*F-B*^*versus ITM2b*^*F-D*^, *p* < 0.001∗∗∗]. *C*, WB of cell surface proteins (B), noncell surface proteins (U) and total lysate (T) isolated from transfected and biotinylated Hela cells. The evidence that the cytosolic αTubulin was not biotinylated indicates that only plasma membrane proteins are biotinylated in these experiments. *C*, quantification of the WBs shown in (*D*) indicates that levels of plasma membrane mBRI2 are significantly lower in cell transfected with mutant BRI2s as compared with wild-type BRI2 [ANOVA summary, F _(2,_ 6) = 51.00, *p* < 0.0002∗∗∗, post-hoc Tukey's multiple comparisons test: *ITM2b*^*F-WT*^*versus ITM2b*^*F-B*^, *p* = 0.0024∗∗, *ITM2b*^*F-WT*^*versus ITM2b*^*F-D*^, *p* = 0.0001∗∗∗, *ITM2b*^*F-B*^*versus ITM2b*^*F-D*^, *p* = 0.0153∗].

To investigate how the British and Danish pathogenic mutations affect BRI2 expression, we transfected HeLa cell with constructs driving expression of either BRI2-Bri23, BRI2-Adan, or BRI2-ABri fused to a Flag-peptide epitope at the NH2-teminus (*ITM2b*^*F-WT*^, *ITM2b*^*F-D*^, and *ITM2b*^*F-B*^, driving expression of F-BRI2-Bri23, F-BRI2-Adan, and F-BRI2-ABri, respectively). The levels of mF-BRI2 were significantly higher in *Itm2b*^*F-WT*^ lysate as compared with *Itm2b*^*F-B*^ and *Itm2b*^*F-D*^ (Fig. 3, *A*–*B*). Also, the levels of F-BRI2-Bri23 were significantly higher compared with F-BRI2-ADan and F-BRI2-ABri (Fig. 3, *A*–*B*). In addition, the ratio of mF-BRI2 to immature F-BRI2 precursor proteins was lower in *Itm2b*^*F-B*^ and *Itm2b*^*F-D*^ as compared with *Itm2b*^*F-WT*^ transfected cells: this difference was highly significant for *Itm2b*^*F-D*^ samples (Fig. 3, *A*–*B*). Although potential differences in transfection efficiencies may be confounding factors, together these data suggest that the British and Danish pathogenic mutations reduce stability and maturation of precursor BRI2-ADan and BRI2-ABri as compared with wild-type BRI2-Bri23.

To further analyze the effect of pathogenic mutation on BRI2 polypeptides trafficking to cell membranes, HeLa cells transfected with BRI2 constructs were surface biotinylated, and the lysates were precipitated with streptavidin (SA) beads to isolate cell membrane proteins (fraction B). Unbound fraction (supernatant fraction U) contains proteins that are not present on the cell membrane and/or exposed to extracellular space. Analysis of the total lysate (T) and fractions B and U confirmed that mBRI2, but not BRI2-Bri23, BRI2-Adan, and BRI2-ABri, was present on the cell surface (fraction B, Fig. 3, *C*–*D*). Given the reduction in mBRI2 levels, *Itm2b*^*F-B*^ and *Itm2b*^*F-D*^ transfected cells presented significantly reduced levels of mBRI2 localized to the plasma membrane compared with *Itm2b*^*F-WT*^ transfected cells (fraction B, Fig. 3, *C*–*D*).

Plasma membrane BRI2 undergoes an additional cleavage by ADAM10 in its ectodomain (10). This cleavage releases a soluble variant of BRI2 and a membrane-bound BRI2 N-terminal fragment (BRI2-NTF). As shown in Figure 3*C*, BRI2-NTFs are visible (and are biotinylated indicating the presence on plasma membranes) in cells transfected with WT BRI2 but not FDD and FBD mutant BRI2, supporting the notion that more mBRI2 is generated from BRI2-Bri23 as compared with BRI2-ADan and BRI2-ABri.

While in synaptoneurosome (Fig. 2*B*) the two pathogenic mutations seem to reduce equally mBri2 levels, there is a more pronounced difference for BRI2-ADan than BRI2-ABri on mBRI2 in HeLa cells, suggesting that the Danish and British mutations may impair maturation of Bri2 in a quantitatively distinct manner in different cell types.

**Figure 4:**
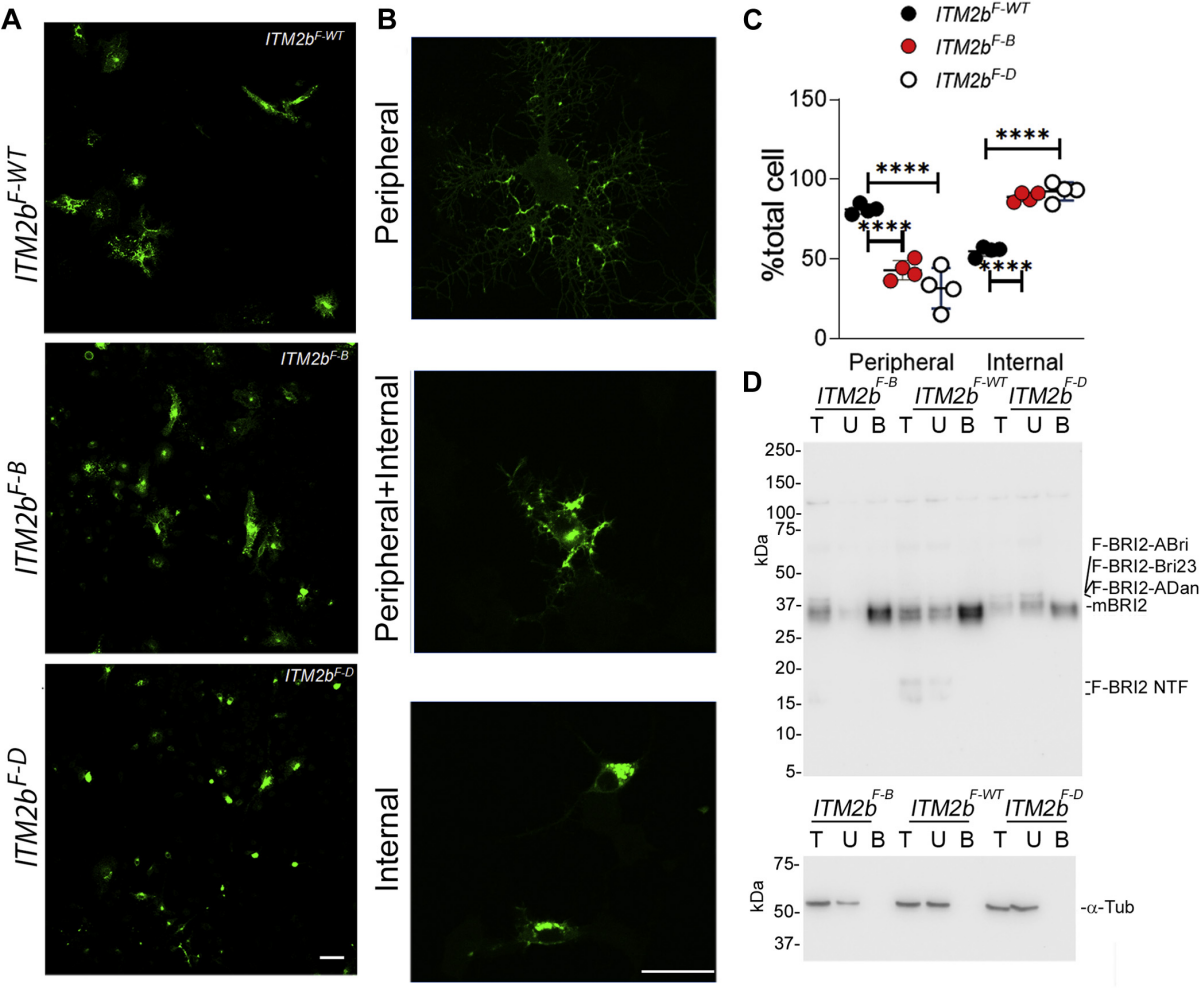
**Distinct subcellular distribution of wild-type and mutant BRI2 forms in N2A transfected cells.** N2A cells were transfected with *Flag*-*ITM2b*^*F-WT*^, *Flag*-*ITM2b*^*F-B*^, and *Flag*-*ITM2b*^*F-D*^ constructs for 24 h and differentiated by 2 μM Retinoic acid for 24 h. *A*, immunocytochemistry staining with anti-Flag M2-FITC, scale bar = 50 μm. *B*, samples of peripheral and internal BRI2 staining used for the quantitative analysis. *C*, quantitative analysis of cell numbers with different BRI2 distributions. Data are represented as mean ± SD and analyzed by ordinary two-way ANOVA followed by post-hoc Tukey's multiple comparisons test when ANOVA showed significant differences [ANOVA summary, F_interaction(2,18)_ = 99.55, *p* < 0.0001, F_Bri2 constructs (1,18)_ = 99.04, *p* < 0.0001, F_distribution type (2, 27)_ = 1.699, *p* = 0.2109, post-hoc Tukey's multiple comparisons test: Peripheral: *ITM2b*^*F-WT*^*versus ITM2b*^*F-B*^, *p* < 0.0001∗∗∗∗, *ITM2b*^*F-WT*^*versus ITM2b*^*F-D*^, *p* < 0.0001∗∗∗∗, *ITM2b*^*F-B*^ *versus ITM2b*^*F-D*^, *p* > 0.05 ns. Internal: *ITM2b*^*F-WT*^*versus ITM2b*^*F-B*^, *p* < 0.0001∗∗∗∗, *ITM2b*^*F-WT*^*versus ITM2b*^*F-D*^, *p* < 0.0001∗∗∗∗, *ITM2b*^*F-B*^*versus ITM2b*^*F-D*^, *p* > 0.05ns]. *D*, biotinylation of transfected N2A cells shows that only mBRI2 traffics to the plasma membrane.

**Figure 5:**
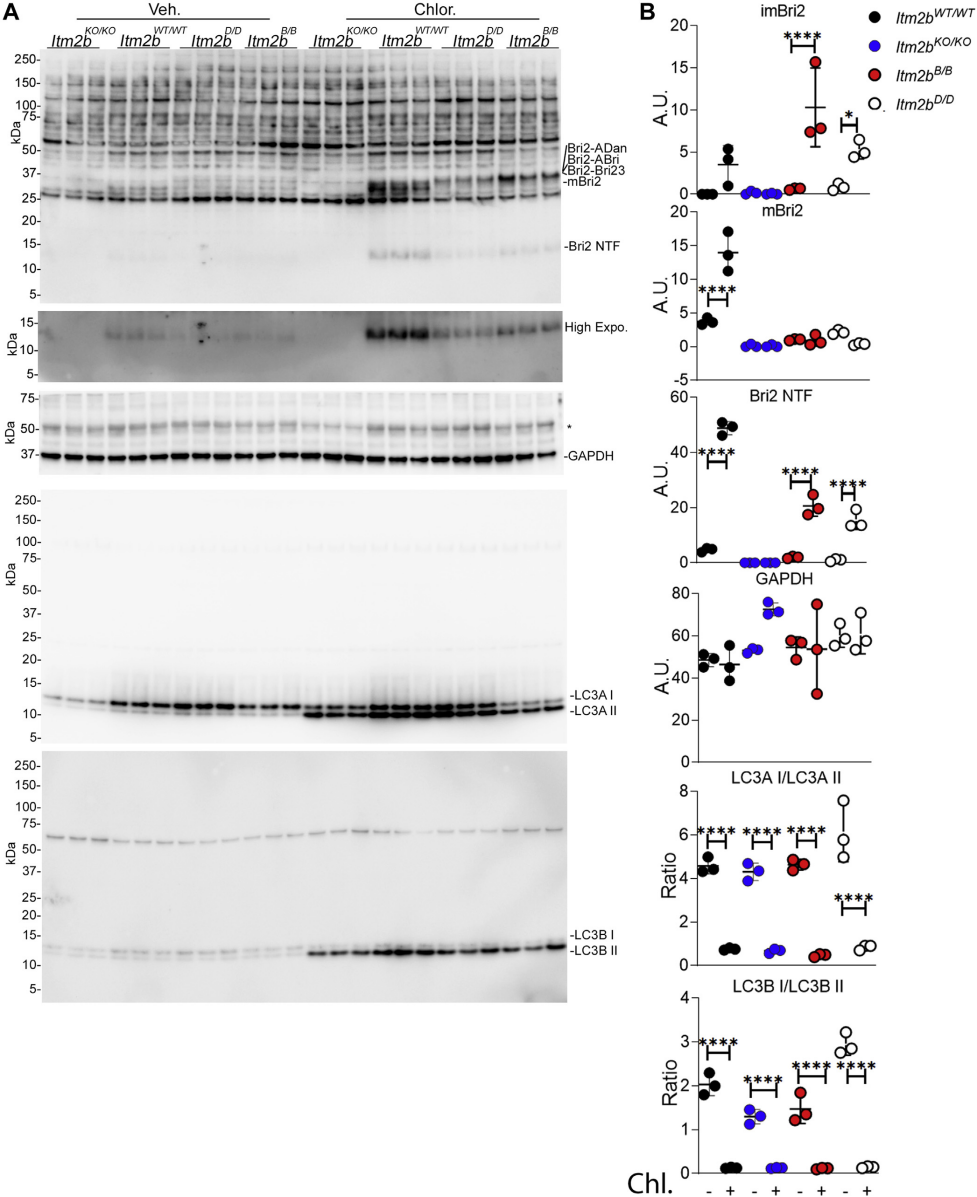
**Distinct degradation pathways of wild-type and mutant Bri2 forms in primary neuron isolated from *Itm2b***^***WT/WT***^**, *Itm2b***^***B/B***^**, and *Itm2b***^***D/D***^**mice.***A*, western immunoblot analysis of primary hippocampal neurons isolated from *Itm2b*^*WT/WT*^, *Itm2b*^*KO/KO*^, *Itm2b*^*B/B*^, and *Itm2b*^*D/D*^ P1 pups treated with 50 μM chloroquine for 18 h. *B*, quantification of imBri2, mBri2, Bri2 NTF, GAPDH, and LC3A/B levels. Data are represented as mean ± SD and analyzed by ordinary two-way ANOVA followed by post-hoc Sidak's multiple comparisons test when ANOVA showed significant differences [ANOVA summary, LC3A: F_interaction(3,16)_ = 2.99, *p* = 0.0621, F_treatment (1, 16)_ = 395, *p* < 0.0001, F_genotype (3, 16)_ = 4.645, *p* = 0.0161, post-hoc Sidak's multiple comparisons test:*Itm2b*^*WT/WT*^*:* veh *versus* Chlor, *p* < 0.0001∗∗∗∗, *Itm2b*^*KO/KO*^*:* veh *versus* Chlor, *p* < 0.0001∗∗∗∗,*Itm2b*^*B/B*^: veh *versus* Chlor, *p* < 0.0001∗∗∗∗, *Itm2b*^*D/D*^: veh *versus* Chlor, *p* < 0.0001∗∗∗∗. LC3B: F_interaction(3,16)_ = 24.53, *p* < 0.0001, F_treatment (1, 16)_ = 599.7, *p* < 0.0001, F_genotype(3, 16)_=25.99, *p* < 0.0001, post-hoc Sidak's multiple comparisons test:*Itm2b*^*WT/WT*^*:* veh *versus* Chlor, *p* < 0.0001∗∗∗∗, *Itm2b*^*KO/KO*^*:* veh *versus* Chlor, *p* < 0.0001∗∗∗∗,*Itm2b*^*B/B*^: veh *versus* Chlor, *p* < 0.0001∗∗∗∗, *Itm2b*^*D/D*^: veh *versus* Chlor, *p* < 0.0001∗∗∗∗. imBri2: F_interaction(3,16)_ = 6.934, *p* = 0.0033, F_treatment (1, 16)_ = 32.33, *p* < 0.0001, F_genotype (3, 16)_ = 8.668, *p* = 0.0012, post-hoc Sidak's multiple comparisons test:*Itm2b*^*WT/WT*^*:* veh *versus* Chlor, *p* = 0.1377 ns, *Itm2b*^*KO/KO*^*:* veh *versus* Chlor, *p* > 0.9999 ns,*Itm2b*^*B/B*^: veh *versus* Chlor, *p* < 0.0001∗∗∗∗, *Itm2b*^*D/D*^: veh *versus* Chlor, *p* = 0.0434∗. mBri2: F_interaction(3,16)_ = 37.01, *p* < 0.0001, F_treatment (1, 16)_ = 21.15, *p* = 0.0003, F_genotype (3, 16)_ = 80.73, *p* < 0.0001, post-hoc Sidak's multiple comparisons test:*Itm2b*^*WT/WT*^*:* veh *versus* Chlor, *p* < 0.0001∗∗∗∗, *Itm2b*^*KO/KO*^*:* veh *versus* Chlor, *p* > 0.9999 ns, *Itm2b*^*B/B*^: veh *versus* Chlor, *p* = 0.9998 ns, *Itm2b*^*D/D*^: veh *versus* Chlor, *p* = 0.2465 ns. Bri2 NTF: F_interaction(3,16)_ = 126.4, *p* < 0.0001, F_treatment (1, 16)_ = 558.6, *p* < 0.0001, F_genotype (3, 16)_ = 187.7, *p* < 0.0001, post-hoc Sidak's multiple comparisons test:*Itm2b*^*WT/WT*^*:* veh *versus* Chlor, *p* < 0.0001∗∗∗∗, *Itm2b*^*KO/KO*^*:* veh *versus* Chlor, *p* > 0.9999 ns, *Itm2b*^*B/B*^: veh *versus* Chlor, *p* < 0.0001∗∗∗∗, *Itm2b*^*D/D*^: veh *versus* Chlor, *p* < 0.0001∗∗∗∗. GAPDH: F_interaction(3,16)_ = 1.825, *p* = 0.1833, F_treatment (1, 16)_ = 1.325, *p* = 0.2667, F_genotype (3, 16)_ = 3.320, *p* = 0.0466, post-hoc Sidak's multiple comparisons test:*Itm2b*^*WT/WT*^*:* veh *versus* Chlor, *p* = 0.9978 ns, *Itm2b*^*KO/KO*^*:* veh *versus* Chlor, *p* = 0.0769 ns, *Itm2b*^*B/B*^: veh *versus* Chlor, *p* > 0.9999 ns, *Itm2b*^*D/D*^: veh *versus* Chlor, *p* > 0.9999 ns.].

**Figure 6:**
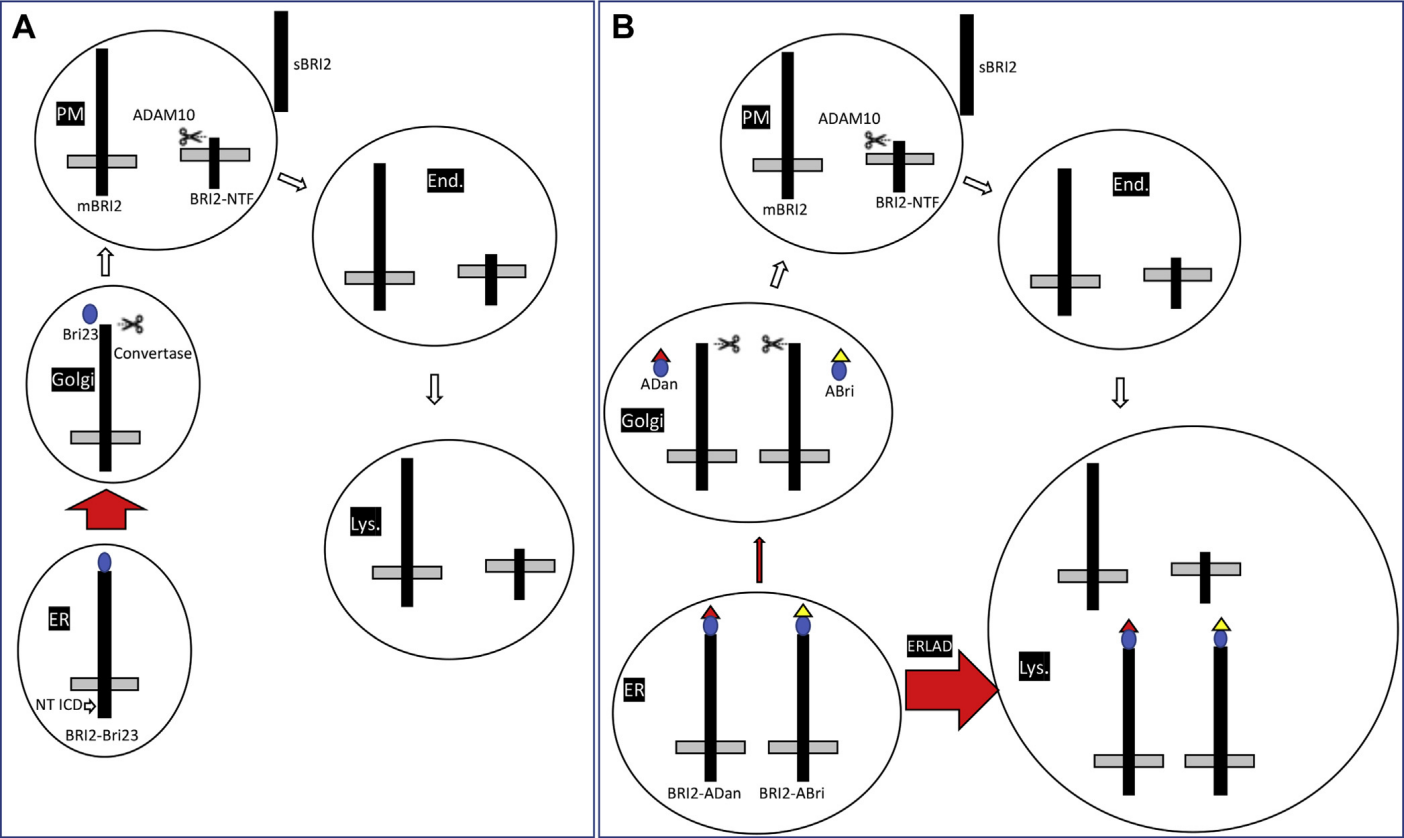
**Modeling wild-type and mutant BRI2 maturation, trafficking, and degradation.***A*, BRI2 is synthesized in the ER as an immature precursors (BRI2-Bri23) that is cleaved in the Golgi apparatus at the C-terminus by proprotein convertase to produce the mature (mBRI2) and the COOH-terminal peptide Bri23. mBRI2 is transported to the plasma membrane where it can undergo an additional cleavage by ADAM10 in its ectodomain. This cleavage releases sBRI2 and the membrane-bound BRI2-NTF. Both mBRI2 and BRI2-NTF are endocytosed and degraded by the lysosomes. *B*, the Danish mutation changes the last amino acid of BRI2 and adds 11 additional amino acids at the COOH-terminus. The British mutations add 11 extra amino acids, which are different from those added by the Danish mutation, at the COOH-terminus as well. A fraction of immature mutant Danish (BRI2-ADan) and British (BRI2-ABri) BRI2 traffic to the Golgi where convertase cleavage generates mBRI2 and two distinct 34 amino acid long peptides, called ADan and ABri, respectively. ADan and ABri can form amyloid fibrils. It is important noting that mBRI2 produced by these pathogenic precursors is identical to the wild-type mBRI2. Large fractions of BRI2-ADan and BRI2-ABri are targeted for ERLAD. It is likely that the ADan and ABri sequences may cause misfolding of BRI2-ADan and BRI2-ABri in the ER.

To better describe trafficking of wild-type and mutant BRI2 proteins, we transfected the F-BRI2 constructs in N2A cells with differentiation and visualized the distribution of BRI2 polypeptides by immunocytochemistry (Fig. 4*A*). This experiment is not used as a proxy for synaptic localization of BRI2. We defined two types of BRI2 staining patterns: identifiable surface membrane staining (compatible with plasma membrane BRI2 localization) *versus* internal staining compatible with BRI2 localization in endoplasmic reticulum, Golgi apparatus, and/or other cytosolic organelles (Fig. 4*B*). Based on these parameters, we quantified a significant reduction of cells with identifiable surface membrane staining but a significant increase in cells with internal staining in *Itm2b*^*F-B*^ and *Itm2b*^*F-D*^ transfected cells compared with *Itm2b*^*F-WT*^ transfected cells (Fig. 4*C*). Consistent with these changes, surface biotinylation of transfected N2A cells showed reduced levels of mBRI2 localized to the plasma membrane in *Itm2b*^*F-B*^ and *Itm2b*^*F-D*^ transfected cells compared with *Itm2b*^*F-WT*^ transfected cells (Fig. 4*C*). Also, in this experiment, BRI2-NTFs are visible and detectable on the plasma membrane in cells transfected with WT BRI2 but not FDD and FBD mutant BRI2

In summary, these observations indicate that pathogenic mutation reduces maturation and plasma membrane localization of mBRI2.

### Increased lysosomal mediated degradation of BRI2-ADan and BRI2-ABri

Transmembrane proteins that enter the ER, but fail to attain the native structure, are rapidly degraded to prevent toxic accumulation of aberrant gene products. These proteins are delivered to endo-lysosome for clearance by autophagic and nonautophagic pathways collectively defined as ER-to-lysosomes-associated degradation (ERLAD) (11). To investigate whether such a mechanism could underlie the maturation deficit and the reduction of mBRI2 caused by the Danish/British mutations, we prepared primary hippocampus neurons from *Itm2b*^*WT/WT*^, *Itm2b*^*B/B*^, and *Itm2b*^*D/D*^ mice. Primary neurons were treated with the lysosomal inhibitor chloroquine.

The first noteworthy observation is that without treatment, the steady-state levels of mBri2, Bri2-NTF as well as the ratio of m/im forms of Bri2 were significantly reduced in *Itm2b*^*B/B*^ and *Itm2b*^*D/D*^ neurons as compared with *Itm2b*^*WT/WT*^ neurons (Fig. 5*A*). This observation parallels what seen in cell lines expressing human wild-type and mutant BRI2. The evidence that chloroquine significantly reduced the LC3A I/LC3A II and LC3B I/LC3B II ratios indicates inhibition of lysosome-mediated degradation. In contrast, GAPDH levels are not changed by chloroquine treatment indicating specificity and lack of toxicity (Fig. 5, *A*–*B*). In neurons derived from *Itm2b*^*WT/WT*^ hippocampi, chloroquine caused a significant and predominant accumulation of mBri2, suggesting that mBri2 may be degraded by lysosome after endocytosis from plasma membranes. In contrast, chloroquine treatment caused exclusively a considerable accumulation of BRI2-ABri and BRI2-ADan in *Itm2b*^*B/B*^ and *Itm2b*^*D/D*^ neurons, respectively (Fig. 5, *A*–*B*).

## Discussion

Our studies suggest that BRI2-ADan and BRI2-ABri may be targeted for ERLAD. The evidence that ADan and ABri peptides tend to aggregate and form amyloid deposits (2, 3) is consistent with the hypothesis that the immature mutant BRI2 proteins, which contain these sequences at the COOH-terminus, may misfold in the ER (Fig. 6). However, a fraction of BRI2-ADan and BRI2-ABri must escape lysosomal degradation and traffic through the *trans*-Golgi where a convertase removes the ADan and ABri peptides, since: 1) ADan and ABri peptides are produced in humans carrying the Danish and British mutation, respectively (2, 3); 2) cells transfected with *ITM2b*^*F-D*^ and *ITM2b*^*F-B*^, *Itm2b*^*B/B*^ and *Itm2b*^*D/D*^ brain lysates and primary neurons contain some mBri2 (Figure 2, Figure 3, Figure 4, Figure 5). In the central nervous system, these maturation anomalies lead to partial loss of mBri2 in synaptoneurosomes (Fig. 2), reduced glutamatergic neurotransmitter release, and AMPAR-mediated responses in *Itm2b*^*B/B*^ and *Itm2b*^*D/D*^ animals (Fig. 1).

Although future studies are needed to assess whether these excitatory synapses dysfunctions described here are pathologically relevant, the evidence presented here is noteworthy because also other genes mutated in Familial forms of dementia, such as *APP*, *PSEN1*, and *PSEN2*, are implicated in glutamatergic synaptic transmission. *APP* modulates glutamate release (12, 13, 14, 15), a function that is altered by a pathogenic *APP* mutation (the Swedish mutation) (15). In addition, long-term potentiation (LTP), a cellular surrogate for memory and memory impairment caused by extracellular Aβ and Tau oligomers are APP-dependent (16). *PSEN1* and *PSEN2* regulate glutamate release by a presynaptic mechanism, a function that is altered by pathogenic mutation (17, 18, 19). Finally, a genetic variant of *TREM2* that increases risk of sporadic AD in humans changes glutamatergic transmission (20) in a rat KI model (21). Our findings that BRI2 also physiologically fine-tunes glutamate transmission with both pre- and postsynaptic mechanisms and that pathogenic mutations dysregulate this function are intriguing and support the hypothesis that defects in excitatory neurotransmitter release may represent a general and convergent mechanism leading to neurodegeneration.

Years ago, we proposed that familial Danish, British, and Alzheimer’s dementias may share a pathogenic sameness. This hypothesis was based on the following evidence: 1) APP and BRI2 physically interact (9, 22, 23, 24); 2) BRI2 alters APP processing (9, 24, 25): 3) APP and APP processing mediate LTP deficits as well as learning and memory deficits of Danish and British KI mice (26, 27, 28, 29, 30, 31). In this context, the evidence that both APP and BRI2 tune excitatory synaptic transmission and that these functions are altered by pathogenic mutations in both APP and BRI2 is intriguing and suggests that APP and BRI2 may functionally interact if in tuning glutamatergic transmission. Future studies will be needed to test this hypothesis.

## Experimental procedures

### Mice and ethics statement

Mice were handled according to the Ethical Guidelines for Treatment of Laboratory Animals of the NIH. The procedures were described and approved by the Institutional Animal Care and Use Committee (IACUC, protocol number PROTO201702513).

### Mice slice preparation

Mice were deeply anesthetized with isoflurane and intracardially perfused with an ice-cold cutting solution containing (in mM) 120 choline chloride, 2.6 KCl, 26 NaHCO_3_, 1.25 NaH_2_PO_4_, 0.5 CaCl_2_, 7 MgCl_2_, 1.3 ascorbic acid, 15 glucose, prebubbled with 95% O_2_/5% CO_2_ for 15 min. The brains were rapidly removed from the skull. Coronal brain slices containing the hippocampal formation (350 μm thick) were prepared in the ice-cold cutting solution bubbled with 95% O_2_/5% CO_2_ using Vibratome VT1200S (Leica Microsystems) and then incubated in an interface chamber in ACSF containing (in mM): 126 NaCl, 3 KCl, 1.2 NaH_2_PO_4_; 1.3 MgCl_2_, 2.4 CaCl_2_, 26 NaHCO_3_, and 10 glucose (at pH 7.3), bubbled with 95% O_2_ and 5% CO_2_ at 30 °C for 1 h, and then kept at room temperature. The hemi-slices were transferred to a recording chamber perfused with ACSF at a flow rate of ∼2 ml/min using a peristaltic pump. Experiments were performed at 28.0 °C ± 0.1 °C.

### Whole-cell electrophysiological recording

Whole-cell recordings in the voltage-clamp mode (−70 mV) were made with patch pipettes containing (in mM): 132.5 Cs-gluconate, 17.5 CsCl, 2 MgCl_2_, 0.5 EGTA, 10 HEPES, 4 ATP, and 5 QX-314, with pH adjusted to 7.3 by CsOH. Patch pipettes (resistance, 8–10 MΩ) were pulled from 1.5 mm thin-walled borosilicate glass (Sutter Instruments, Novato, CA) on a horizontal puller (model P-97; Sutter Instruments, Novato, CA). Basal synaptic responses were evoked at 0.05 Hz by electrical stimulation of the Schaffer collateral afferents using concentric bipolar electrodes. CA1 neurons were viewed under upright microscopy (FN-1, Nikon Instruments, Melville, NY) and recorded with Axopatch-700B amplifier (Molecular Devices, San Jose, CA). Data were low-pass filtered at 2 kHz and acquired at 5 to 10 kHz. The series resistance (Rs) was consistently monitored during recording in case of reseal of ruptured membrane. Cells with Rs >20 MΩ or with Rs deviated by >20% from initial values were excluded from analysis. Excitatory postsynaptic currents (EPSCs) were recorded in ACSF containing 15 μM bicuculline methiodide to block GABA-A receptors. The stimulation intensity was adjusted to evoke EPSCs that were 40% of the maximal evoked amplitudes (“test intensity”). 5 to 10 min after membrane rupture, EPSCs were recorded for 7 min at a test stimulation intensity that produced currents of ∼40% maximum. For recording of paired-pulse ratio (PPR), paired-pulse stimuli with 50 ms or 200 ms interpulse interval were given. The PPR was calculated as the ratio of the second EPSC amplitude to the first. For recording of AMPA/NMDA ratio, the membrane potential was firstly held at −70 mV to record only AMPAR current for 20 sweeps with 20 s intervals. Then the membrane potential was turned to +40 mV to record NMDAR current for 20 sweeps with perfusion of 5 μM NBQX to block AMPAR. Mini EPSCs were recorded by maintaining neurons at −70 mV with ACSF containing 1 μM TTX and 15 μM bicuculline methiodide to block action potentials and GABA-A receptors, respectively. mEPSCs were recorded for 5 to 10 min for analysis. Data were collected and analyzed using the Axopatch 700B amplifiers and pCLAMP10 software (Molecular Devices), and mEPSCs are analyzed using mini Analysis Program.

### Mice brain preparation and synaptoneurosome preparation

Mouse brain fractionations were prepared as follows. Brains were homogenized (w/v = 100 mg tissue/1 ml buffer) in buffer A (5 mM HEPES, 1 Mm MgCl_2_, 0.5 mM CaCl_2_, PH7.4) supplemented with protease/phosphatase inhibitor (Thermo Fisher Scientific 78440) on ice. Homogenate was centrifuged at 1400*g* for 10 min. The supernatant was collected and labeled as S1 fraction. S1 was centrifuged at 13,800*g* for 10 min to generate pellet. The pellet was resuspended in 300ul buffer B (0.32 M sucrose, 6 mM Tris, pH 8.0), layered a discontinuous sucrose gradient (0.85, 1.0 and 1.2 M sucrose, 6 mM Tris, pH 8.0), and centrifuged at 83,000*g* for 2 h. SP fraction was present as a white band at the 1.0/1.2 M interface. The SP fraction was lysed with Buffer T (5% Triton X-100, 6 mM Tris, pH 8.0) to achieve final Triton-X 100 concentration to 0.5%. After 15 min shaking on ice, samples were centrifuged at 20,000*g* for 20 min. The separated supernatant was collected and labeled as Triton soluble (SPs) fraction. The pellet was resuspended in 50 ml Buffer T and was labeled as Triton insoluble (SPi) fraction.

### scRNA-seq analysis

Full project data was downloaded from the Mouse Development Atlas (32). The full expression values and metadata were downloaded in loom format and imported to Seurat for additional analyses. Cells expression values were normalized using sctransform and clustered following the standard workflow (33). Cluster markers were transferred from the development atlas to ensure parity with cell classifications. For cell coexpression of molecules, a normalized value of 1 was considered positive for the query gene of interest, and the expression of Itm2b was pulled for each positive cell for comparative visualization.

### Plasmids

Constructs of Flag-human full-length BRI2 (*ITM2b*^*F-WT*^) in pcDNA3.1 was previously described (24). Flag-FBD mutant BRI2 (*ITM2b*^*F-B*^) and Flag-FDD mutant BRI2 (*ITM2b*^*F-D*^) were both obtained by mutagenesis of *ITM2b*^*F-WT*^. *ITM2b*^*F-B*^ expressed the extra C-terminal 11 amino acids long sequence “RTVKKNIIEEN”. In *ITM2b*^*F-D*^ the last amino acid, a Ser (S), is replaced by “FNLFLNSQEKHY.”

### Cell culture and transfection

Neuro-2A (N2A) cells (ATCC CCL-131) and Hela cells (ATCC CCL-2) were maintained in Eagle's Minimum Essential Medium (EMEM) (Gibco 11095-098) supplemented with 10% fetal bovine serum and Antibiotic-Antimycotic (Gibco 15240112). Both Hela cells and N2A cells were transfected with indicated plasmids *via* Fugene kit (Promega, E2311) as previously described (34, 35, 36).

### Immunocytochemistry and cell counting

The N2A cells were plated on coverslips precoated with poly L-lysine (Sigma, P4707). Twenty-four hours after transfection, N2A cells were differentiated by 0.1% FBS and 2 mM retinoic acid for 24 h. Differentiated N2A cells were washed with PBS, fixed by 4% PFA, permeabilized with 0.1% Triton X-100 in PBS, blocked with 10% BSA in PBS, incubated with Anti-Flag M2 FITC (Sigma, F4049), and visualized on confocal microscope (Nikon A1R). For cell counting, we replicated n = 4 independent transfection and took ten nonoverlapped images for each transfection. An average of 250 cells were counted for each transfection.

### Biotinylation, streptavidin precipitation

Biotinylation experiments were performed as described (9). Twenty-four hours after transfection, HeLa cells or N2A cells were washed three times with cold PBS plus Ca^2+^ and Mg^2+^ (PBS-CM) and labeled for 30 min on ice in 0.5 mg/ml sulfo-NHS-SS-biotin (Pierce) dissolved in PBS-CM. Media was removed by washing three times with PBS-CM containing 0.1% BSA. The cells were lysed in RIPA buffer (10 mM Tris-Cl, 1 mM EDTA, 0.5 mM EGTA, 1% Triton X-100, 0.1% SDS, 140 mM NaCl). The lysates were cleared by centrifuging at 20,000*g* for 10 min and were mixed with streptavidin agarose beads (Sigma S1638). After collecting unbound lysate (U), the beads were washed four times with the RIPA buffer and were boiled in 2× SDS buffer (B). Comparable volume of the samples was subjected to Western immunoblot. The streptavidin precipitants correspond to five times of total lysates or unbound proteins.

### Primary hippocampus neuron culture

Mouse hippocampus neurons were derived from *Itm2b*^*WT/WT*^, *Itm2b*^*KO/KO*^, *Itm2b*^*B/B*^, and *Itm2b*^*D/D*^ postnatal day 0 mouse pups. Briefly, after the removal of adherent meninges, the hippocampi were collected in dissection media (HBSS w/o Mg and Ca, 1 mM sodium pyruvate, 0.1%glucose, 0.01 M HEPES). The hippocampi were dissected into single cell suspension with trituration following 15 min 37 °C 0.25% trypsin digestion. Cells were subsequently applied 0.1% Dnase (Sigma, dn25) and enriched in plating media (BME, 10% FBS, 0.09% Glucose, 1 mM Sodium Pyruvate, 2 mM Glutamine, 1× Pen/Strep). Cells were passed through Falcon 70 μm nylon cell strainer and counted with Hemacytometer. 300k dissociated cells were plated in each single well in Poly L lysine pretreated 12-well-plate in 1 ml of maintenance media (Neurobasal media, 1× B-27, 2 mM glutamine, 1× Pen/Strep). Cells were refeed with 0.5 ml of maintenance media every 2 days. Cells were used for further treatment at DIV 14.

### Pharmacological treatment and sample preparation

Primary neurons were treated with 50 mM Chloroquine (Cell signaling, 14774s) or same amount of PBS (Veh) for 18 h. Treated cell were washed with PBS and lysed in RIPA buffer with protease/phosphatase inhibitor for 15 min on ice. Lysed samples were centrifuged at full speed for 15 min. The supernatants were mix with LDS buffer supplemented with 10% β-mercapto-ethanol. Samples were boiled for 2 min before loading to a gel.

### Western immunoblot analysis

Protein content quantified by Bradford analysis. Ten micrograms from each fraction was brought to 10 μl with PBS and LDS buffer supplemented with 10% β-mercapto-ethanol and 8 M Urea. Denatured samples were loaded on a 4 to 12% Bis-Tris polyacrylamide gel (Bio-Rad, 3450125) and run at constant voltage (140 V). Proteins were transferred onto nitrocellulose membrane at 25 V for 7 min using *Trans*-blot Turbo system (Bio-Rad) and visualized by red Ponceau staining. Membranes were blocked with 5% nonfat dry milk (Bio-Rad, 1706404), washed with PBS/Tween-20 (0.05%), and applied primary antibody (see list and information about the dilution used in Table 1), which is diluted in blocking solution (Thermo Fisher Scientific, 37573) overnight at 4 °C. Membranes were washed 3 × 10 min and subsequently against by either HRP linked antimouse (SouthernBiotech, OB103105) or anti-rabbit (1:1 mix of SouthernBiotech, OB405005 and Cell Signaling Technology, 7074) at 1:1000 dilution for 45 min with shaking in room temperature. Membranes were developed with West Dura ECL reagent (Thermo Fisher Scientific, PI34076) and visualized on a ChemiDoc MP Imaging System (Bio-Rad). Signal intensity was quantified with Image Lab software (Bio-Rad). Data were analyzed using Prism software and represented as mean ± SD.

**Table 1:** List of antibodies used

Antibody	Brand	Cat. #	Host	Type	Usage	note
α-tubulin	Sigma-Aldrich	T6199	Mouse	monoclonal	1:1000	
Bri2-N terminus	D'Adamio's lab		Rabbit	polyclonal	6 μg/ml	Raised against peptide "AQKEAKKDEPKSSE"
NMDA R2B	Cell Signaling Technology	4212	Rabbit	monoclonal	1:1000	
PSD95	Cell Signaling Technology	2507	Rabbit	polyclonal	1:1000	
Vamp2	Synaptic Systems	104,211	Mouse	monoclonal	1:1000	
Flag-hrp	Sigma-Aldrich	A8592	Mouse	monoclonal	1:1000	
Flag-fitc	Sigma-Aldrich	F4049	Mouse	monoclonal	1:1000	
LC3A	Cell Signaling Technology	4599	Rabbit	monoclonal	1:1000	
LC3B	Cell Signaling Technology	2775	Rabbit	polyclonal	1:1000	
GAPDH	Sigma-Aldrich	G9545	Rabbit	polyclonal	1:1000	

### Statistics

All the experiments mentioned in the paper were analyzed by one-way ANOVA or two-way ANOVA by indication. Data showing statistical significance by one-way ANOVA or two-way ANOVA were subsequently analyzed either by Tukey's multiple comparisons test or by Sidak's multiple comparisons. All statistical analyses were performed using Prism 8 (GraphPad) software.

## Data availability

The data sets used and/or analyzed during the current study are available from the corresponding author on reasonable request.

## Conflicts of interest

The authors declare that they have no conflicts of interest with the contents of this article.
